# Assessing nitrogen dynamics model and the role of artificial lagoon in effluent loading of shrimp farms in Gomishan wetland, southern Caspian Sea

**DOI:** 10.1038/s41598-022-26458-7

**Published:** 2022-12-26

**Authors:** Fateme Ghodrati, Rasoul Ghorbani, Naser Agh, Aliakbar Hedayati, Rahmat Naddafi, Ali Jalali, Fakhrie Shiroudmirzaei

**Affiliations:** 1 Faculty of Fisheries and Environmental Sciences, Gorgan University of Agricultural Sciences and Natural Resources, Gorgān, Iran; 2 Artemia and Aquaculture Research Iinstitute, Urmia University, Urmia, Iran; 3 Department of Aquatic Resources, Swedish University of Agricultural Sciences, 75007 Uppsala, Sweden; 4 Faculty of Science, Engineering and Built Environment, Deakin University, Geelong, VIC Australia; 5 Ph.D. Graduate of Fisheries, Faculty of Fisheries and Environmental Sciences, Gorgan University of Agricultural Sciences and Natural Resources, Gorgān, Iran

**Keywords:** Biological techniques, Environmental sciences

## Abstract

Shrimp farming increases the nutrients, especially nitrogen in the water resources reducing water quality. This study was conducted to evaluate the nitrogen dynamics in white shrimp (*Litopenaeus vannamei*) farms and the role of artificial lagoon (24 ha) in reducing nitrogen levels in Gomishan coastal wetland, the eastern Caspian Sea. The results indicated that at the end of the 4-month breeding period, the amounts of nitrogen and phosphorus introduced into Gomishan wetland were calculated as to 220.157 and 39 tons, respectively in a breeding area covering 830 hectares. Nitrogen values (based on nitrate) calculated based on the relationship between the basin and the discharge of the outlet channel of the site at the time of complete emptying of the farms, were calculated to be approximately 121.8 tons per breeding time that it had an important role in eliminating about 45% of nutrient pollution and reducing the concentration of dissolved nitrogen. Moreover, nitrogen isotopic trace was observed in shrimp samples, in similar levels in the samples of both shrimp pond and lagoon, which emphasizes the role of feeding from natural food, especially benthic fauna. Overall, due to the decline of Caspian Sea water level, Gomishan coastal wetland is drying, and the output of shrimp farms is currenly the only source supplying water for the wetland. Hence, appropriate management strategies could minimize the amounts of nutrients into the natural water whilst aiding wetland’s survaival.

## Introduction

In recent years, aquaculture, especially in developing countries, has become increasingly widespread. Aquaculture is one of the human activities with its uncontrolled development imposing negative effects on the environment^1^. Coastal ecosystems are affected by environmental effects created by excess nutrients from natural processes and anthropogenic activities adjacent to watersheds^2^.

In coastal areas, high organic and nutrient loadings generated from feed wastage, excretion and faecal productions are directly discharged into the environment, therefore, concerns have been expressed that the interaction between a farm and its environment could result in destructive responses with adverse effects on the coastal and marine ecosystems as well as on the farm itself^3^. Therefore, understanding the nutrient budget of fish farms is useful in mariculture development and management, since knowledge on the loading and forms of nutrients from various sources enable appropriate measure to be devised for the sustainable development of auquacultur industry^3^.

Pollutants and stressors affect water quality and alter its physicochemical and biological properties^4^. Therefore, before these effluents have a negative effect on the sustainability of aquaculture activities, their negative effects need to be evaluated^5^. The effluents of nutrient-rich shrimp pond include phosphate, nitrogen compounds, and organic matter, which increase the concentration of phytoplankton^6^. The amount and type of feed given to shrimp are the main source of nutrients in effluents^7^. Previous studies showed that only 20 to 40 percent of the nitrogen in food is absorbed by shrimp^8^, and the rest enters the environment and consumed by other organisms such as phytoplankton and bacteria. Nitrogen in breeding systems can be used both as a nutrient and as a toxic contaminant to damage the breeding system. Hence, it is an important parameter in the dynamics of breeding systems^9^. Castillo-Soriao et al., in providing a model for nitrogen dynamics from the beginning of cultivation to harvest under low salinity and with no water change in shrimp ponds showed that in these systems, due to the retention of nitrogen in the water, shrimp has a lower feed conversion ratio compared to rearing farming systems^10^.

Stable isotope analysis (SIA) is used as a useful tool in studying population dynamics including determining the diet and nutritional status of the ecosystem^11^. The amount of material transported to rivers and canals, as well as the amount and quality of food, may vary. Therefore, it is expected that a spatial and temporal change in the stable isotope values (δ13C and δ15N) of consumers is reflected. Human activities severly alter the nitrogen cycle in terrestrial and aquatic ecosystems, increasing the nitrogen load in many rivers^12^. Stable isotopic analyses are becoming widespread as a tool for studies of community structure and ecosystem function^13^. In trophic studies, heavier isotopes of any given element increase in abundance compared with lighter isotopes, through the process of isotope discrimination. Early laboratory studies showed that for carbon (C) the isotopic ratio values of consumers are usually similar to those of their diets. The use of stable isotopic techniques to study animal diets and trophic levels requires a priori estimates of discrimination factors (∆^13^C and ∆^15^ N, also called fractionation factors), which are the differences in isotopic composition between an animal and its diet^14^. Post^13^ showed that the mean trophic fractionation of d15N is 3.4‰and of d13C is 0.4‰, and that both, even though variable, are widely applicable.

Wetlands are complex and valuable ecosystems that are involved in flood control, groundwater quality improvement, coastal protection and conservation, fish production, tourism, wildlife sanctuary, genetic, plant and animal resources, and regional climate change, and other values ​​play an important role^15^. Due to recent droughts and increased human activities in Gomishan wetland, such as the expansion of Gomishan shrimp farms, the level of pollutants is increasing in the wetland, whilst, due to the reduction of the Caspian Sea water level and its disconnection with Gomishan wetland, this wetland is drying. Proper management of this wetland with high ecological value becomes more necessary. Therefore, the aim of this study was to evaluate the nitrogen dynamics of white shrimp (*Litopenaeus vannamei*) farms and the role of artificial lagoon in reducing the level of nitrogen in Gomishan coastal wetland in the southern Caspian Sea.

## Material and methods

**Figure 1:**
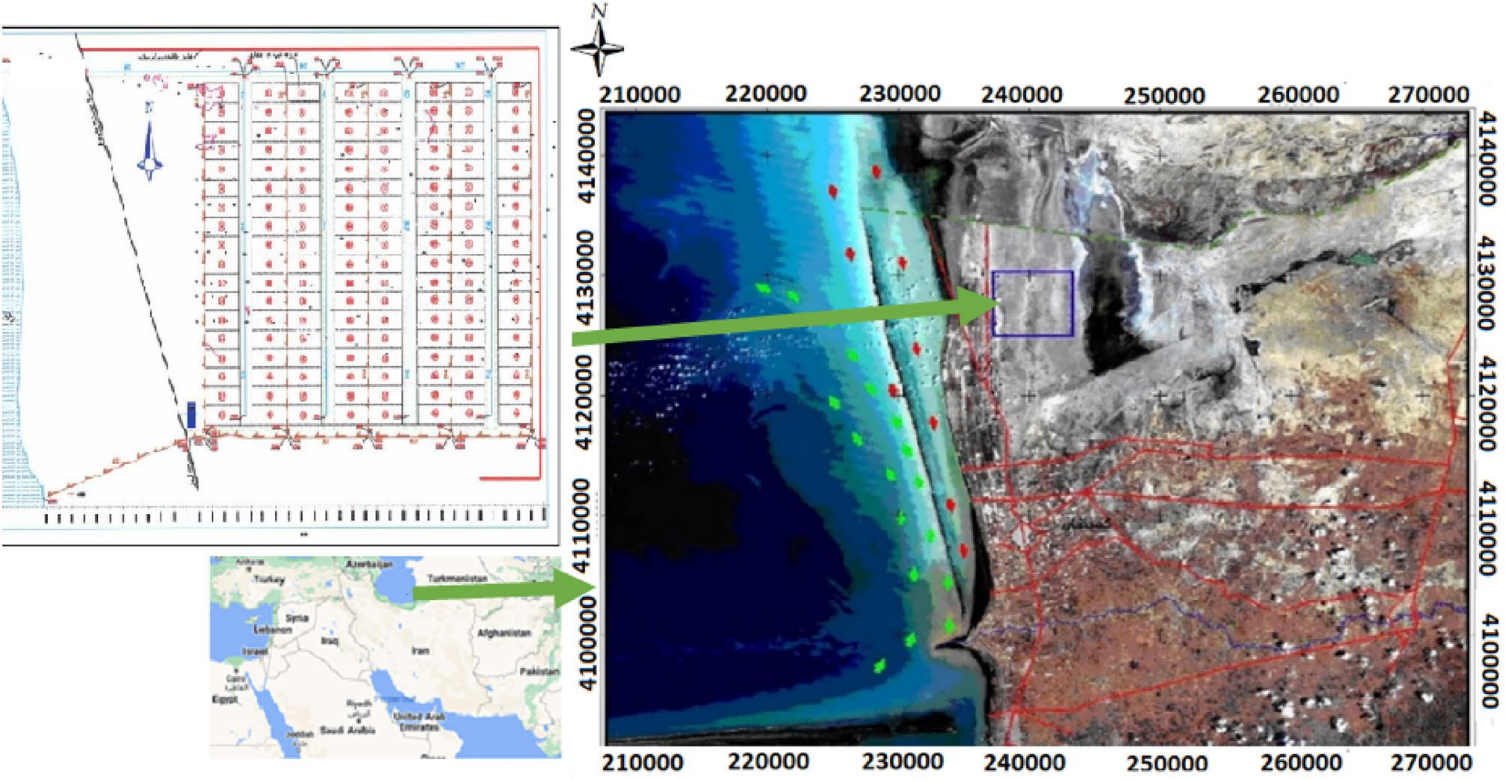
Location of Gomishan Wetland and Gomishan Shrimp Cultivation Complex^17^ cited it^18^.

Gomishan wetland (37° 9′ 9″ and 37° 21′ 20″ N and 53° 54′ 34″ and 53° 58′ 54″ E) covers the areas of about 20,000 hectares^16^, with depth of about 1 m (Riyazi, 2001). Shrimp farms are located near the wetland in the southeast of the Caspian Sea^17^ (Fig. 1).

The water required for the Gomishan shrimp site is supplied directly from the Caspian Sea through a main canal (MC). The effluent from the pools returns to Gomishan wetland through five drainage channels (MD) through each main drainage channel (MD) (velocity = 0.14 m/s, depth = 0.4, width = 76.7 m) and after crossing lagoon (24 ha) to increase water retention time and the opportunity for initial water self-purification^17^. During the 4-month breeding period, due to the high evaporation of the water and to compensate for the water loss, about 10% of the water is changed. A simplified, conceptual, nutrient mass balance model was proposed to derive the level of nitrogen and phosphorus discharged from a hypothetical pond. The model was intended to estimate the approximate level of N and P added to the environment for every ton of shrimp produced, based on various assumptions on feed loss, FCR, N and P content of feeds and shrimp and various nutrient dynamics within the pond^19^.

In addition, based on the total N content and daily discharge values of the outlet channel (late July to late October), the amount of nitrogen loading the Gomishan wetland was estimated using Web Based Load Calculation software using LOADEST. Water nitrogen data were obtained from the online monitoring system located under the bridge (200 m after the lagoon) daily at 11 o’clock.

In this research, a mathematical model was prepared using STELLA 8™ software. This model describes the nitrogen flow in the aquatic environment through the interaction of shrimp farms with the environment. Moreover, the stable isotope values of nitrogen and carbon were checked in the feed plate used in feeding vanami shrimp, benthos, pond shrimp samples, shrimp samples in the outlet channel, mullet, carp, gambusia and goby in the outlet channel and in the Caspian Sea, in order to track the amounts of nitrogen and carbon isotopic in the environment and the food network. In the laboratory, sampled macroinvertebrates and the dorsal muscle tissue of fish were frozen until further analyses. The frozen samples were freeze-dried for 48 h. Then, samples from each site were grounded into a powder and stored in small glass vials for later isotope analysis. Furthermore, dorsal muscle tissue from fish from each site was grounded and placed in glass vials. The aim for analyzing these samples was to compare isotope values and its positions of different food web components at the Gomishan shrimp site. All glass vials were labeled and stored over silica gel desiccant. The samples were analyzed according to previous studies^20^. Nitrogen and carbon contents as well as nitrogen (δ15N) and carbon (δ13C) stable isotopic composition were analyzed using an Isotope Ratio Mass Spectrometer interfaced with an Elemental Analyzer (EA-IRMS).

## Results

Based on a simple and conceptual model for nitrogen and phosphorus depleted for the shrimp farm, and using various assumptions about the low frequency of macrobenthos (due to the use of netting to prevent their entry), nutrient dynamics in the farming system was calculated: nitrogen and phosphorus in the feed 6.5% and 1.4%, respectively, and in shrimp, 3% and 1%. In the market weight of 15–16 g and total biomass (about 3 tons) at the end of the 4-month breeding period, the hypothetical model with the nitrogen and phosphorus entried into Gomishan wetland, was equivalent to 220.157 tons and 39 tons (with a total breeding area of 830 hectares/240,000 ind/ha in 2018, FCR = 1.29) and 99.41 tons and 10.7 tons (with a total area of 1251/100,000 ind/ha hectares in 2021, FCR = 1.22) (Table 1).

**Table 1:** Parametes measured (per hectar) in Gomishan shrimp farms.

Year (Gomishan shrimp site)	2018 (830 ha)			2021 (1251 ha)		
Parameters/day	50	80	110	50	80	110
N_pl_/ha(× 1000)	195	185	185	81	77	77
Weight (gr)	0.6	8.5	15.48	0.6	8.5	15.5
Biomass (ton)	0.117	1.574	2.87	0.0487	0.656	1.196
Feed (ton)	0.094	2.283	4.162	0.039	0.918	1.615
N feed (ton)	0.006	0.148	0.271	0.0025	0.06	0.105
P feed (ton)	0.0013	0.032	0.058	0.00054	0.0129	0.0226
Unconsumed feed (ton)	0.037	0.685	1.249	0.0039	0.092	0.161
Unconsumed feed N (ton)	0.0024	0.045	0.081	0.00025	0.006	0.0105
Unconsumed feed P (ton)	0.0005	0.0096	0.0175	0.000055	0.0013	0.0023
Meet N (ton)	0.0035	0.0472	0.0861	0.0015	0.0197	0.036
Meet P (toon)	0.0012	0.0157	0.0287	0.00049	0.0066	0.012
Excretion N through faeces (ton)	0.0026	0.101	0.184	0.0011	0.04	0.069
Excretion P through faeces	0.00014	0.0162	0.03	0.00006	0.0063	0.011
N_inorganic_ loads in the culture site (ton)	0.005	0.146	0.266	0.0013	0.046	0.08
P_inorganic_ loads in the culture site (ton)	0.00066	0.0258	0.047	0.0001	0.0076	0.013
N_inorganic_ (total) (ton)	220.16			99.41		
P_inorganic_ (total) (ton)	39			10.7		

Due to the presence of white spot virus, the density decreased to 100,000 ind/ha.

**Figure 2:**
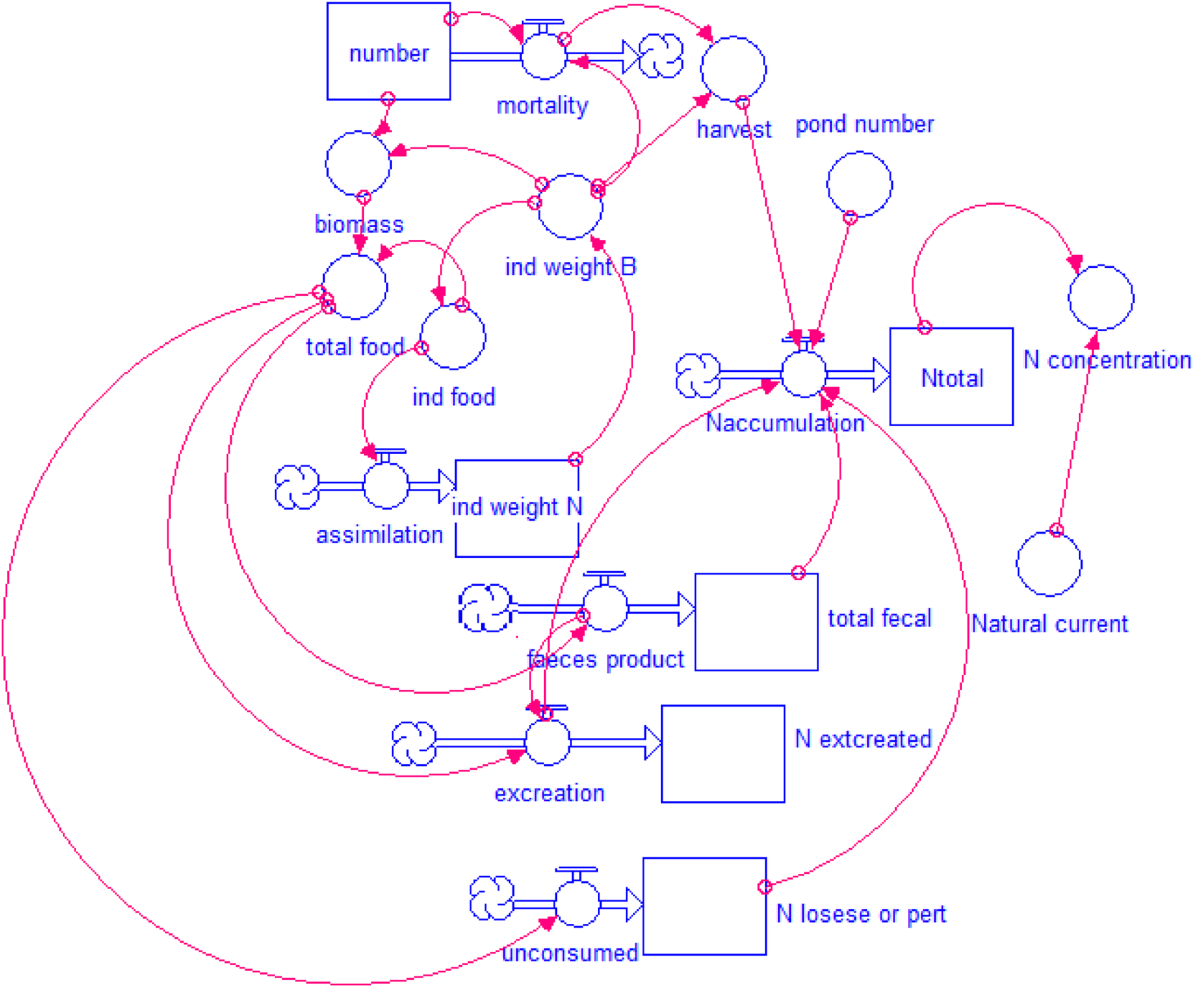
Nitrogen dynamics model from shrimp farming.

The amount of nitrogen in shrimp farming was calculated through daily flow velocity and density based on the mathematical formulas in Stella model (Fig. 2).

$$ \begin{aligned} & {\text{ind}}\_{\text{weight}}\_{\text{N}}\left( {\text{t}} \right) = {\text{ind}}\_{\text{weight}}\_{\text{N}}\left( {{\text{t}} - {\text{dt}}} \right) + \left( {{\text{assimilation}}} \right)*{\text{dt}} \\ & {\text{INIT}}\;{\text{ind}}\_{\text{weight}}\_{\text{N}} = .0{27} \\ \end{aligned} $$

INFLOWS:

$$ \begin{aligned} & {\text{assimilation}} = {\text{ind}}\_{\text{food}}*0.{16}*0.{3}*0.{9}*0.{5} \\ & {\text{Ntotal}}\left( {\text{t}} \right) = {\text{Ntotal}}\left( {{\text{t}} - {\text{dt}}} \right) + \left( {{\text{Naccumulation}}} \right)*{\text{dt}} \\ & {\text{INIT Ntotal }} = \, 0 \\ \end{aligned} $$

INFLOWS:

$$ \begin{aligned} & {\text{Naccumulation}} = {\text{IF}}\left( {{\text{harvest}} = 0} \right){\text{THEN}}\left( {{\text{pond}}\_{\text{number}}*\left( {{\text{excreation}}} \right)} \right){\text{ELSE}}\left( {{\text{pond}}\_{\text{number}}*\left( {{\text{excreation}} + {\text{total}}\_{\text{fecal}} + {\text{N}}\_{\text{losese}}\_{\text{or}}\_{\text{pert}}} \right)} \right) \\ & {\text{number}}\left( {\text{t}} \right) = {\text{number}}\left( {{\text{t}} - {\text{dt}}} \right) + \left( { - {\text{mortality}}} \right)*{\text{dt}} \\ & {\text{INIT number}} = {24}0000 \\ \end{aligned} $$

OUTFLOWS:

$$ \begin{aligned} & {\text{mortality}} = {\text{IF}}\left( {{\text{ind}}\_{\text{weight}}\_{\text{B}} < {2}0} \right){\text{THEN}}\left( {0.0{1}*{\text{number}}} \right){\text{ELSE}}\left( {{\text{number}}} \right) \\ & {\text{N}}\_{\text{extcreated}}\left( {\text{t}} \right) = {\text{N}}\_{\text{extcreated}}\left( {{\text{t}} - {\text{dt}}} \right) + \left( {{\text{excreation}}} \right)*{\text{dt}} \\ & {\text{INIT}}\;{\text{N}}\_{\text{extcreated}} = .0{77}*{\text{total}}\_{\text{food}} \\ \end{aligned} $$

INFLOWS:

$$ \begin{aligned} & {\text{excreation }} = {\text{ total}}\_{\text{food}}*0.{3}*.{16}*.{85} - \left( {{\text{faeces}}\_{\text{product}} + {\text{total}}\_{\text{food}}*0.{3}*0.{16}*0.{85}*0.{25}} \right) \\ & {\text{N}}\_{\text{losese}}\_{\text{or}}\_{\text{pert}}\left( {\text{t}} \right) = {\text{N}}\_{\text{losese}}\_{\text{or}}\_{\text{pert}}\left( {{\text{t}} - {\text{dt}}} \right) + \left( {{\text{unconsumed}}} \right)*{\text{dt}} \\ & {\text{INIT}}\;{\text{N}}\_{\text{losese}}\_{\text{or}}\_{\text{pert}} = 0 \\ \end{aligned} $$

INFLOWS:

$$ \begin{aligned} & {\text{unconsumed}} = {\text{total}}\_{\text{food}}*0.{3}*0.{16}*0.{15} \\ & {\text{total}}\_{\text{fecal}}\left( {\text{t}} \right) = {\text{total}}\_{\text{fecal}}\left( {{\text{t}} - {\text{dt}}} \right) + \left( {{\text{faeces}}\_{\text{product}}} \right)*{\text{dt}} \\ & {\text{INIT}}\;{\text{total}}\_{\text{fecal}} = 0.{26}*{\text{ind}}\_{\text{food}} \\ \end{aligned} $$

INFLOWS:

$$ \begin{aligned} & {\text{faeces}}\_{\text{product}} = {\text{total}}\_{\text{food}}*.{3}*.{16}*.{85}*.{1} \\ & {\text{biomass}} = {\text{number}}*{\text{ind}}\_{\text{weight}}\_{\text{B}}*.{2} \\ & {\text{harvest}} = {\text{IF}}\left( {{\text{ind}}\_{\text{weight}}\_{\text{B}} < {1}0} \right){\text{THEN}}\left( 0 \right){\text{ELSE}}\left( {{\text{mortality}}*{\text{ind}}\_{\text{weight}}\_{\text{B}}} \right) \\ & {\text{ind}}\_{\text{food}} = {\text{ind}}\_{\text{weight}}\_{\text{B}}*.0{3} \\ & {\text{ind}}\_{\text{weight}}\_{\text{B}} = {\text{ind}}\_{\text{weight}}\_{\text{N}}/0.0{285} \\ & {\text{Natural}}\_{\text{current}} = {14}0 \\ & {\text{N}}\_{\text{concentration}} = \left( {{\text{Ntotal}}/{\text{Natural}}\_{\text{current}}/{1}000} \right) \\ & {\text{pond}}\_{\text{number}} = {83}0 \\ & {\text{total}}\_{\text{food}} = {\text{biomass}}*{\text{ind}}\_{\text{food}}*{1}.{287} \\ \end{aligned} $$

**Figure 3:**
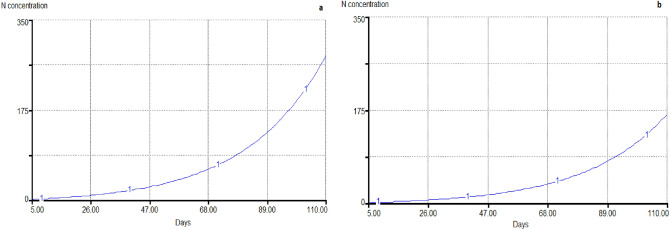
Estimated amounts of nitrogen outlet from Gomishan shrimp farms (**a** 830 ha; **b** 1251 ha).

According to the Fig. 3, based on density of 240,000 ind/ha in 2018, the average water flow velocity in the outlet channel was equal to 140 L per second, maximum nitrogen concentration equal to 280.9 mg/l and based on density 100,000 ind/ha, it was predicted to be 166.8 mg/l (Fig. 3).

**Figure 4:**
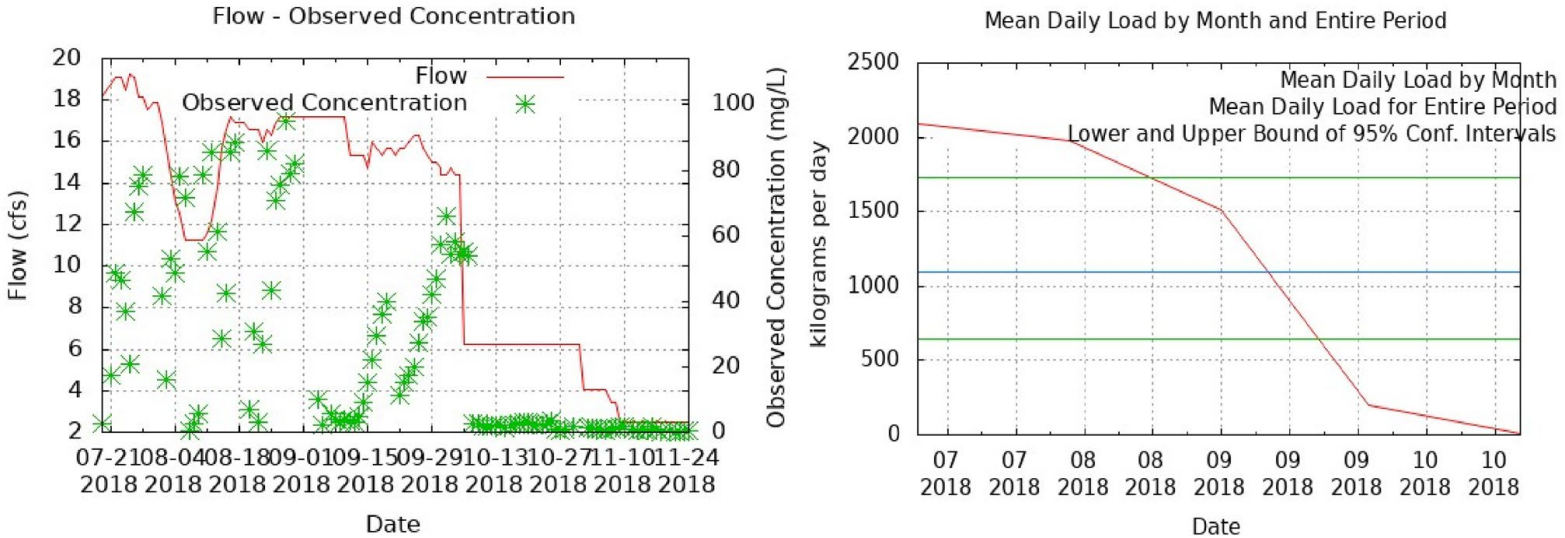
Average nitrogen loading in the outlet channel of Gomishan shrimp farms.

Nitrogen values (based on nitrate) calculated based on the relationship between the basin and the discharge of the outlet channel of the site at the time of complete emptying of the farms (using Web Based Load Calculation software using LOADEST) were calculated to be approximately 398.58 tons per year (121.8 ton per breeding time (Fig. 4).

In comparison, stable isotope samples, the highest amount of isotopic nitrogen was obtained in goby and the lowest amount was obtained in food plates and gammaridae. Isotopic nitrogen levels in food plates were not significantly different from those in gammaride, white shrimp and Gambusia but were significantly different from mullet, common carp and goby (*P* < 0.05). Also, the lowest amount of isotopic carbon was in the food plate and it was significantly less than other biological samples. But no significant difference was observed among the studied biotic samples (*P* > 0.05) (Table 2).

**Table 2:** Stable isotope values of nitrogen and carbon in indicator organisms in pond, lagoon and outlet canal.

Sample		C13		N15
Food plate	− 22.1	Unit (‰)	4.28	Unit (‰)
Gammaridae	− 16.9 ± 1.9*a	5.2 (↑ ΔP)	4.6 ± 0.49b	0.32 (↑ ΔP)
Shrimp	− 17.3 ± 1.2*a	4.8 (↑ ΔP)	7.42 ± 2.11ab	3.14 (↑ ΔP)
Gambusia	− 17.4 ± 0.5*a	− 0.5 (↓ ΔGa)	7.94 ± 3.8ab	3.34 (↑ ΔGa)
Mullet	− 18.7 ± 0.1*a	− 1.8 (↓ ΔGa)	6.43 ± 0.22*b	1.83 (↑ ΔGa)
Goby	− 18.3 ± 1.4*a	− 0.9 (↓ ΔGm)	10.24 ± 0.23*a	2.3 (↑ ΔGm)
Carp	− 18.6 ± 0.9*a	− 1.7 (↓ ΔGa)	7.69 ± 0.45*ab	3.09 (↑ ΔGa)

P, Plate; Ga, Gammaridae; Gm: Gambusia.

Different letters mean significant at 0.05.

**Figure 5:**
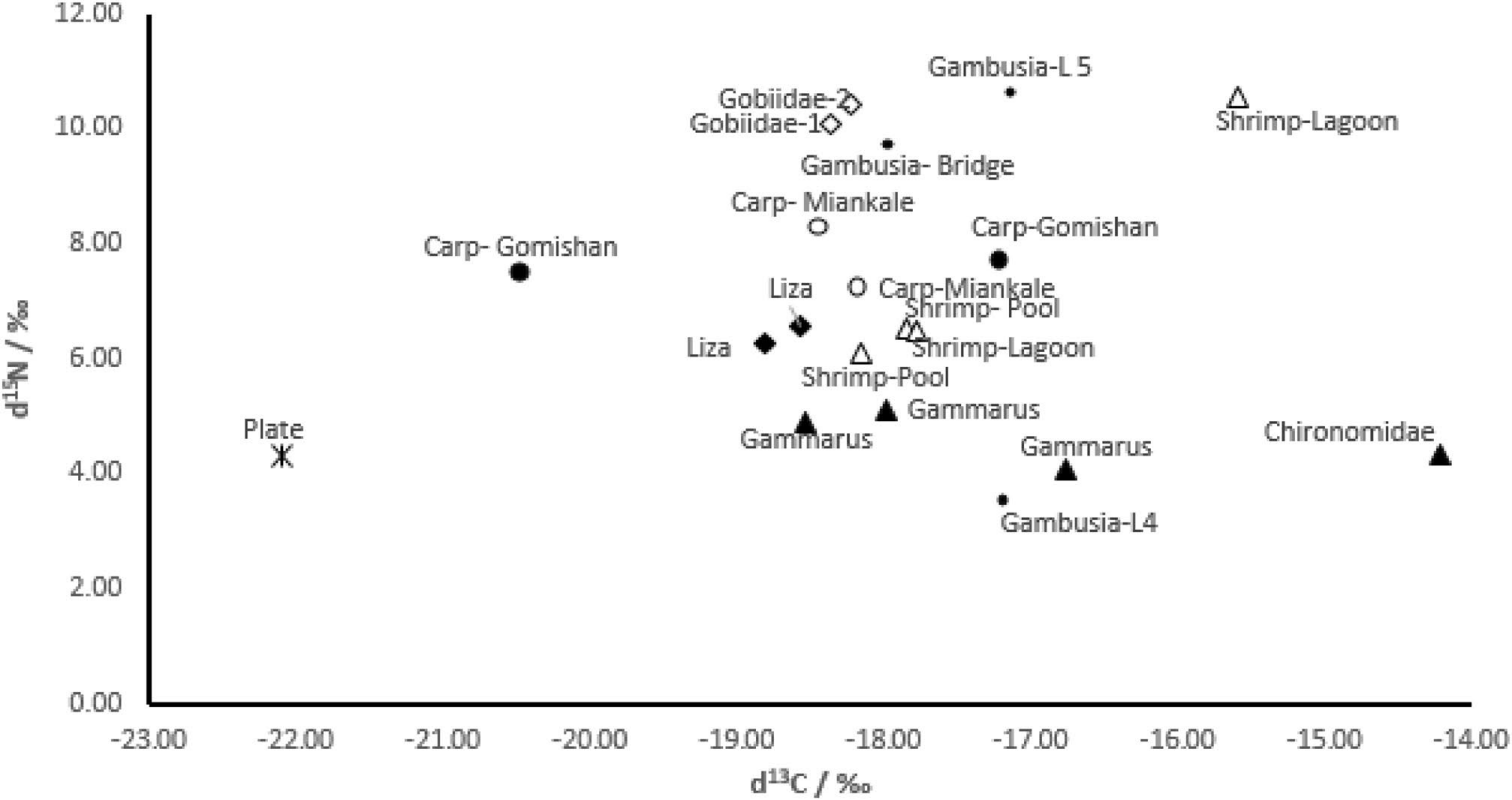
Isotopic position of the food network in the outlet channel of Gomishan site and the southeastern part of the Caspian Sea.

The isotopic values of carbon versus nitrogen in the diagram show the shrimp samples in the pond and some of the lagoon samples were close, while some of the shrimp samples in the lagoon are far apart. This is also being in isotopic carbon content in gammaridae and Gambusia (Fig. 5).

## Discussion

Recent development of aquaculture industry, despite positive advantages, has caused problems such as the entry of various pollutants into the environment^21^. Unfortunately, in many countries, the uncontrolled development of shrimp farming near coastal waters has been associated with many environmental problems^22^. In Sri Lanka, as an instance, salt marshes at the Puttalam/ Mundal estuary, especially in the floodplain Mioya, have been severely threatened since the emergence of shrimp farms^23^. Adverse effects of shrimp farms lead to the processes of mangrove degradation, natural drainage change, saline infiltration and habitat degradation of aquatic species^3,24^. These effluents include high concentrations of nutrients, suspended solids, and organic matter^25^, which reduce dissolved oxygen and increase water turbidity.

The exchanging water can change the morphological balance of coastal systems^26^. Factors such as location, management and existing density^25^ affect the quality of shrimp farm effluent and reduce its level. Nutrients of shrimp farms also indicated to be the most common sources of contamination in coastal estuaries and lagoon in northeastern Brazil^2,27^. In this respect, the results of this study showed that the amount of nitrogen loading from Gomishan shrimp breeding site after temporary storage in lagoon in 2018, with a storage density of 240 thousand per hectare in a total area of 830 hectares equivalent to 220.16 tons and the estimated amount of phosphorus was 39 tons. With the spread of the white spot virus, breeding at the Gomishan site has almost stopped. However, after three years, 1251 hectares of farms started operating in 2021 with a minimum storage of 100 thousand per hectare and the estimated amounts of nitrogen and phosphorus were equal to 99.4 tons and 16.12 tons, respectively.

Also, in estimating the amount of nitrogen loaded using the concentration of nitrogen in canal water, the amount of 878.92 tons per year (264.9 ton per breeding time) was estimated, which indicates the removal of approximately 45% of the amount of nitrogen entering the lagoon and the effective role of the existing lagoon (the activity of microorganisms such as decomposing bacteria, phytoplanktons, macrophytes and other nitrogen fixers) in removing it from the water, and, the concentration of nitrogen entering the Gamishan wetland was greatly reduced.

Mayer et al.^12^ believe that due to mass balance, less than 30% of human nitrogen enters the sea to large basins and through runoff and rivers, and more than 70% of nitrogen are stored and decomposed in watersheds, which confirms the results of this study.

In the study of Chaikaew et al.^28^ it was estimated that the total nitrogen outlet from the shrimp pond was 107.8 kg, which is 35.5% of the effluent. 17% becomes shrimp product, 46.4% accumulates in sediments, the remaining 1.2 is released through the atmosphere. The total phosphorus outlet is 178.4 kg, of which 43.1% is converted into effluent, 53.5% is accumulated in sediments, 0.7% in shrimp. 2.8% remains unused. The presence of high density of aquatic plants along the existing lagoon as well as in the lateral parts of the outlet channel and the sludge depth of about 10 to 15 cm indicates the absorption of high amounts of nitrogen or sediment. Fourooghifard et al.^29^ showed that breeding Vanami shrimp and the alga *Gracilaria corticata* can increase total shrimp production, reduce the amount of nitrogen and phosphorus in both water and sediment, and improve water quality. Considering the average water flow velocity in the outlet channel equal to 140 L per second, the maximum nitrogen concentration of 280.9 mg/l was expected, which by comparing the maximum nitrogen concentration values using an online monitoring device, the highest values at 11 o’clock, it was about 190 mg/l, which shows the role of the lagoon as well as the water velocity as well as the width of the outlet channel in reducing the concentration of water-soluble nitrogen.

In addition to the hours of the day, Shiroud Mirzaei et al.^18^ pointed out the importance of the breeding day in increasing the amount of nitrogen, so that on the nintieth day of breeding, the amount of nitrogen will increase suddenly. Thus, it can be said that the amount of nitrogen in this day has a more effective role in determining the average amount of nitrogen. In this way, by controlling feeding on this critical day, the amount of nitrogen output can also be controlled.

Stable isotope levels of carbon and nitrogen are widely used in the study of food network fluctuations in aquatic ecosystems^30^. In comparison with stable isotopes, the highest amount of isotopic nitrogen in goby was due to the carnivorous diet, which seems to be compared to its prey groups such as mullet juveniles and gambosia, according to the results of Post^13^ which was expected to increase about 3 ‰ in isotopic nitrogen, but its isotopic carbon content decreased by 1 to 2 ‰, relative to prey. In farmed shrimp, which are predominantly fed on feed plates, isotopic nitrogen levels above 3 ‰, and isotopic carbon values increased by about 5‰. The results indicate that food plates are rich in nitrogen relative to carbon.

Isotopic nitrogen levels increased by less than 0.5 ‰in the gammaridae, isotopic carbon levels increased by about 5 ‰ like shrimp, while food plates had the lowest isotopic carbon content. On the other hand, due to the fact that the gammaridae samples from the outlet channel shows a very small increase in isotopic nitrogen levels, it indicates that they either rarely feed unused food plates in the effluent sediments or that most of the available nitrogen in the effluent of unused plates as well as the excreta of farmed shrimp, it is absorbed by aquatic plants, lagoon and outlet canals. High levels of isotopic carbon in aquatic groups (compared to platets) may be related to the type of diet and the use of natural pray and plant detritus. Gammarida also appears to play an important role in feeding shrimp and other aquatic groups and is the main source of feeding in the outlet canal and lagoon. Isotopic values ​​of shrimp samples in the pool and some lagoon samples were placed close together in the diagram, while some shrimp caught in the lagoon had very high amounts of nitrogen and isotopic carbon. It is possible that some shrimps have just entered the lagoon from the pond, while some specimens have been in the lagoon longer. Due to the benthic diet of shrimp and feeding on gammaridae, it seems that even inside the pond (with low frequency), they play an important role in feeding shrimp.

No overlap was observed among goby due to their carnivorous diet with other groups and also between mullet with other groups due to the detritus diet, while there was food overlap among the other groups.

## Conclusion

In general, based on the modeling results, the amount of nitrogen in the effluent of shrimp farms and, consequently, in Gomishan wetland increases following the storage of the initial number of postlarvae shrimp. On the other hand, Gomishan wetland, as the final destination for the discharge of shrimp farms, due to the decreasing of the Caspian Sea water level and also the increase of evaporation due to global warming, is exposing a decrease in water depth every year; So that in almost years, most part of wetland has dried up.

This problem is exacerbated when some managers believe that the release of shrimp farming effluent may be able to prevent the drying of the Gomishan wetland, prevent the spread of fine dust, and even increase shrimp production as a way to maintain wetland is known; While the amount of nitrogen released to it through the effluent of shrimp farms indicates a serious environmental problem, they do not pay attention to the consequences of increasing nutrients. However, it should be noted that the only factor that currently keeps Gomishan Wetland from drying out so far is the entry of shrimp farm effluent, which can be turned with management strategies including make of lagoon and treatment of incoming effluent.

## Data Availability

The datasets used and/or analysed during the current study available from the corresponding author on reasonable request.
